# Effects of Implementing an ICU Discharge Readiness Checklist on *Patient Safety Culture*: A Quasi-Experimental Research Study

**DOI:** 10.3390/healthcare13070816

**Published:** 2025-04-03

**Authors:** Vanja Vončina, Hana Brborović, Ognjen Brborović, Alka Makovšek, Jadranka Pavičić Šarić

**Affiliations:** 1 Department of Anesthesiology, Intensive Care Medicine and Pain Management, Merkur Clinical Hospital, Zajčeva 19, 10000 Zagreb, Croatia; vcrnica@gmail.com (V.V.); jadranka.pavicic.saric@kb-merkur.hr (J.P.Š.); 2 School of Medicine, University of Zagreb, Šalata 3, 10000 Zagreb, Croatia; 3 Department of Environmental and Occupational Health and Sports Medicine, Andrija Štampar School of Public Health, School of Medicine, University of Zagreb, Rockefellerova 4, 10000 Zagreb, Croatia; 4 Department of Social Medicine and Health Care Organization, Andrija Štampar School of Public Health, School of Medicine, University of Zagreb, Rockefellerova 4, 10000 Zagreb, Croatia; obrborov@snz.hr; 5 Clinic of Anesthesiology, University Hospital Centre Zagreb, Mije Kišpatića 12, 10000 Zagreb, Croatia; alka.makovsek@gmail.com; 6 Department of Anesthesiology, Reanimatology, Intensive Care and Emergency Medicine, Faculty of Medicine, University of Rijeka, Braće Branchetta 20, 51000 Rijeka, Croatia

**Keywords:** *patient safety culture*, patient safety climate, patient safety, safety culture, discharge readiness checklist, patient handover, intensive care unit, ICU throughput

## Abstract

**Background:** Discharging patients from intensive care units (ICUs) poses significant risks for adverse events (AEs), contributing to hospital morbidity and mortality. To mitigate premature transitioning, an ICU discharge readiness checklist (ICU-DRC) was developed. Enhanced *patient safety culture* (PSC) is crucial for reducing AEs and improving outcomes. Given the pressing need to enhance PSC in ICUs, this study evaluates the impact of ICU-DRC implementation on PSC, aiming to address a critical gap in quality improvement. **Methods:** A prospective quasi-experimental study assessed PSC before and after a year-long ICU-DRC intervention at Merkur Clinical Hospital in Zagreb, Croatia. Healthcare providers from two distinct ICUs participated voluntarily in the *Hospital Survey on Patient Safety Culture*. The surgical ICU, where the intervention was applied, employed 106 providers, while the medical ICU, which did not implement the intervention, had 42 providers. **Results:** Initial response rates were 58% for the intervention group and 45% for the control group, with post-intervention rates of 53% and 48%, respectively. The ICU-DRC was utilized with a fidelity of 65.7%. Due to the non-normal distribution found for most variables, non-parametric analytical procedures were applied. In baseline measurements, the control group outperformed the intervention group in three out of fourteen PSC dimensions. Post-intervention, PSC scores in the intervention group significantly improved in one dimension, whereas three dimensions in the control group showed significant declines, resulting in superior PSC outcomes for four dimensions in the intervention group during the second measurement. **Conclusions:** Applying the ICU-DRC as an isolated safety intervention aimed at optimizing ICU patient throughput resulted in observable patterns of improvement in several PSC dimensions, with statistically significant changes in specific areas.

## 1. Introduction

While the seminal work *To err is human* [1] ushered in an era of profound academic and practical interest in patient safety, a quarter of a century later, *adverse events* (*AE*s) remain a significant burden, both in terms of patient harm and economic costs [2,3,4,5,6,7,8]. Estimated exposure to AEs varies from 12% [5] to 25% [6], or 132 million patients annually, leading to 2.6 million deaths [6]. Also, 60% of deaths from curable diseases are estimated to result from unsafe medical practices and low-quality healthcare [7]. Furthermore, as treating the consequences of unsafe medical practices hinders healthcare accessibility, AEs could become one of the main obstacles to achieving universal healthcare coverage [9].

As patients treated in an *intensive care unit (ICU)* are those who are by definition the sickest and in need of the most sophisticated care [10], the incidence of inadvertent failures in care is unsurprisingly the highest here [5,11,12,13], reaching a staggering 52.1% [14]. A higher incidence of ICU AEs can necessitate additional life-sustaining interventions [15], is clearly linked with increased length of stay (LOS) both in the ICU and the hospital, and contributes to a higher mortality rate [16].

Handoffs and transitions are particularly sensitive moments in the treatment process [17], as they are connected to a higher probability of AEs [18], medication errors [19], decreased patient satisfaction with treatment [20], increased costs of care [21], and increased mortality [22]. An estimated 15% of handoffs and transitions will result in an AE [23].

In the context of an ICU, an increase in demand could lead to capacity strain, in turn causing both early discharge and delaying necessary treatment for patients who are waiting for admission [24,25]. Premature discharge increases the risk of readmission and related mortality [26], while delayed discharge may result in harm from over-treatment and place an additional burden on a scarce resource [24,27].

In 2022, Hiller et al. introduced a set of criteria designed to assess the discharge readiness of adult patients in ICUs. This framework serves as both a prompt for early evaluation of a patient’s discharge status and a checklist aimed at preventing premature discharge. The criteria can be formalized to serve as an *ICU discharge readiness checklist (ICU-DRC)* [28].

Checklists have long been established as valuable tools in improving *patient safety* in various clinical settings [29,30,31,32,33,34], yet their full impact depends on whether or not they can be applied consistently and whether or not they will lead to a meaningful change in *patient safety culture (PSC)*, typically defined as “*The product of individual and group values, attitudes, perceptions, competencies and patterns of behavior that determine the commitment to, and the style and proficiency of, an organization’s health and safety management*”. Or, as Pronovost and Sexton phrased it: “*In essence, culture is “the way we do things around here”, whereby the word “here” refers not to the hospital, but rather to a particular work unit* [35,36,37,38,39]”.

The sustained interest in measuring PSC [35,36,37] is due to *safety culture* being perceived as both a proxy outcome for quality of care and a sine qua non of quality improvement [4,6,9,40]. A recent scoping review confirmed this by showing an association between increased PSC and reduced AE rates [40].

The necessity for a structured discharge process from the ICU, which involves applying a set of criteria to create a *discharge readiness checklist*, has been well documented in the existing literature [17,41,42,43] and endorsed by relevant professional societies [44,45,46]. Evidence supports the effectiveness of similar checklists in various medical fields [18,20,21,47,48,49,50]. However, the World Health Organization Surgical Safety Checklist (WHO SSC) [51] did not improve *patient safety culture* until it was integrated into a broader national safety initiative [33,52,53]. The *discharge checklist* proposed by Hiller et al. in 2022 is relatively novel and, as far as we are aware, has not yet been evaluated for its efficacy in any capacity. To our knowledge, no previous study has evaluated whether applying a structured *ICU discharge readiness checklist* can directly enhance PSC as measured by standardized tools like the *Hospital Survey on Patient Safety Culture* (HSOPSC). This represents a significant knowledge gap that our study aims to fill, providing novel insights into how structured discharge protocols might improve *safety culture* within healthcare institutions.

## 2. Materials and Methods

### 2.1. Design

**Figure 1:**
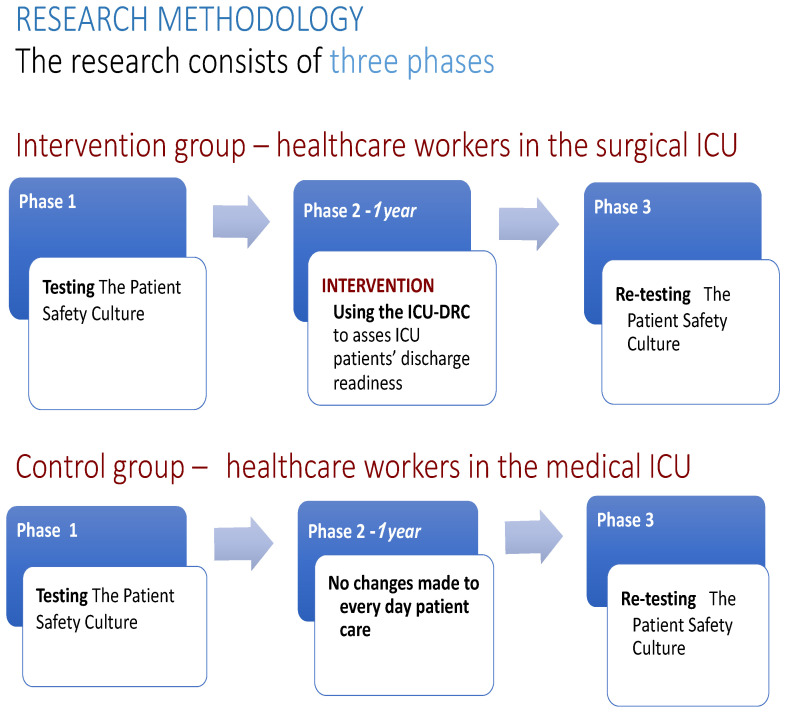
The research timeline.

We conducted a quasi-experimental, controlled intervention study at Merkur Clinical Hospital in Zagreb, Croatia, to investigate *patient safety culture (PSC)* using the validated Croatian version of the *Hospital Survey on Patient Safety Culture (HOSPSC)* [37,54]. The study analyzed PSC both prior to and following the implementation of the *ICU discharge readiness checklist (ICU-DRC)*, which was introduced as a mandatory part of the standard discharge process in the intervention ICU. The intervention group consisted of healthcare providers working in the ICU of the anesthesiology, intensive care, and pain management departments *(surgical ICU)*, while the control group included healthcare providers working in the ICU of the Clinic of Internal Medicine *(medical ICU)*; both units operate within the same hospital, sharing a common institutional *safety culture*. This quasi-experimental design was chosen as it is often the most feasible approach in healthcare settings, where randomizing healthcare workers between units is impractical, likely unethical, and would certainly confound the baseline results of *patient safety culture*. The shared hospital context provided a more comparable setting than ICUs in different hospitals. The research timeline is depicted in Figure 1. The HOSPSC was initially distributed to healthcare providers in both ICUs in December 2022. As of 1 January 2023, the ICU-DRC was implemented in the surgical ICU, while the discharge process remained unchanged in the medical ICU. A second PSC assessment was conducted in January 2024, after one year of ICU-DRC implementation.

### 2.2. Population

The study was performed in a tertiary teaching hospital, Merkur Clinical Hospital, in Zagreb, Croatia, where patients in need of intensive care are treated within two independent ICUs. All healthcare providers—doctors, nurses, and physical therapists—working in either ICU were invited to participate in the survey.

As part of the Department of Anesthesiology, Intensive Care Medicine and Pain Management, *the surgical ICU*—cares for patients who require perioperative intensive care, i.e., patients with acute failure of one or more vital organs who must be stabilized immediately prior to or after elective or emergency surgery. The *surgical ICU* has a capacity of 14 beds and a total of 106 healthcare providers—40 doctors, 64 nurses, and 2 physical therapists. In 2022, 1567 patients were admitted to this unit, while there were 1663 admissions in 2023. Healthcare providers in the *surgical ICU* were invited to participate as the *intervention group (IG)*, as the intervention—implementation of the ICU-DRC—was being carried out in that intensive care unit.

The *medical ICU* is organizationally a part of the cardiology department of the Clinic for Internal Medicine. This ICU typically treats patients in need of acute vital organ support that cannot be provided in the lower levels of care when the acute (multiple) vital organ dysfunction/failure is not a consequence of trauma or surgery, nor does it require immediate surgical treatment. A total of 42 healthcare providers—18 doctors, 23 nurses, and 1 physical therapist—provide care to patients occupying 12 beds. In 2022, 882 patients were admitted to this unit, while there were 867 admissions in 2023. The *medical ICU* healthcare providers were invited to serve as the *control group (CG)* in this study, as the discharge process was not deliberately changed for patients treated in this ICU.

As the study was conducted as a part of PhD thesis research, approval was obtained both from the Ethical Committee of Merkur Clinical Hospital (protocol code: Reg. no.: 03/1-6070, date of approval 18 July 2022) and the University of Zagreb School of Medicine (protocol code: Reg. no.: 380-59-10106-23-111/12 Class: 641-01/23-02/01, date of approval 30 January 2023). The study was conducted in accordance with the Declaration of Helsinki.

Informed consent forms were distributed together with printed surveys but were collected in separate boxes to ensure anonymity.

### 2.3. Materials

The HOSPSC was developed by the US Agency of Healthcare Research and Quality [37,54]. It consists of 42 questions, which address 12 safety culture dimensions (*Teamwork Within Units, Supervisor/Manager Expectations, Organizational Learning–Continuous Improvement, Management Support for Patient Safety, Overall Perceptions of Patient Safety, Feedback Communication About Errors, Communication Openness, Frequency of Events Reported, Teamwork Across Units, Staffing, Handoffs and Transitions*, and *Nonpunitive Response to Errors*) and two single-item measures (*Patient Safety Grade* and *Number of Events Reported*). A table describing how each dimension is formed is presented in Appendix A, Table A1 [37,54,55,56]. Numerical values are appointed to each dimension and single-item measures, as previously described in the literature [37,54,55,56]. As Brborović et al. described: “The 42 items of the HSOPSC mostly use the 5-point Likert response scale of agreement (‘Strongly disagree’ to ‘Strongly agree’) or frequency (‘Never’ to ‘Always’). Most of the questionnaire’s items asked respondents to answer using 5-point response categories in terms of agreement (‘Strongly agree’, ‘Agree’, ‘Neither’, ‘Disagree’, ‘Strongly disagree’) or frequency (‘Always’, ‘Most of the time’, ‘Sometimes’, ‘Rarely’, ‘Never’). Three of the 12 PSC composites use the frequency response option (‘Feedback and communication about error’, ‘Communication openness’, and ‘Frequency of events reported’), while the other nine composites use the agreement response option. The Safety Grade section requires each participant to assign a grade (on the traditional A to F scale) to patient safety observed by their operational unit (a one-item measure scored from 4 (A) to 0 (F), a higher score indicating a higher patient safety level). Adverse event reports are assessed on a one-item scale that asks: “In the past 12 months, how many adverse event reports have you filled out and submitted?”. Response categories span from no adverse event reports (0), 1 to 2 (1), 3 to 5 (2), 6 to 10 (3), 11 to 20 (4), and ≥21 reports (5). The dimension results can be interpreted either as a percentage or a value, where a number lower than 3 equals weakness, three equals a neutral state, and above 3 equals strength” [54].

The Croatian version of the HOSPSC has been in use for over 10 years and is deemed adequate for secondary and tertiary levels of care [54,55,56,57,58,59,60,61,62,63,64]. The psychometric properties of the questionnaire have been reported as satisfactory at the individual, unit, and hospital levels [65,66,67], and this survey was found to be the broadest in scope among a plethora of instruments currently used to measure PSC [36].

The checklist for assessing patient readiness for discharge from the ICU is based on the criteria published by Hiller et al. in 2022 [28]. It consists of 28 criteria divided into 9 groups: *Respiratory System*, *Cardiovascular System*, *Central Nervous System*, *Pain*, *Urogenital System*, *Fluid Loss and Drainage*, *Medication and Nutrition*, *Patient’s Diagnosis*, *Prognosis*, *and Preferences*, and *Institution-specific Criteria*. The full list of criteria, as published originally, is available in Appendix A, Table A2 [28]. To the best of our knowledge, this checklist has not yet been tested in clinical practice in relation to *patient safety culture*.

### 2.4. Outcome Measures

Following our aim of assessing the influence of introducing the ICU-DRC on PSC, we specified three outcomes to be addressed with this study:

(1) Compare PSC measured by the adapted Croatian version of the HOSPSC between the *intervention* and *control groups* prior to the introduction of the ICU-DRC.
(2) Assess the fidelity in the use of the ICU-DRC for the *intervention group*.
(3) Assess the difference in changes in PSC measured in both the *intervention group* and *control group* after the intervention was conducted for a period of one year.

### 2.5. Statistical Analysis

Statistical analysis was performed using SPSS Statistics version 25 (IBM Corp., Armonk, NY, USA). Quantitative data distribution was analyzed for normality using the Smirnov–Kolmogorov and Shapiro–Wilk tests, which showed a non-normal distribution for most variables. Therefore, non-parametric analytical procedures were used in the analysis.

Distributions are described by conventional measures of descriptive statistics (median (M), minimum (min) and maximum (max) values, and interquartile range (IQR)) and then analyzed using the Mann–Whitney U test. To determine effect size, r values were calculated (r = Z/√N) for all statistically significant results and interpreted using Cohen’s established benchmarks for interpretation (small: 0.1, medium: 0.3, large: 0.5). The distribution of qualitative data was analyzed using Fisher’s exact test. Statistical significance was set at *p* < 0.05 along with Bonferroni correction, adjusting the significance threshold to *p* < 0.05/k, i.e., *p* < 0.025.

## 3. Results

Normality was assessed using both the Kolmogorov–Smirnov (with Lilliefors correction) and Shapiro–Wilk tests for the *intervention group* (n = 56). The Shapiro–Wilk test indicated non-normal distributions for *Supervisor/Manager Expectations* (W = 0.952, *p* = 0.025), *Communication Openness* (W = 0.953, *p* = 0.028), *Nonpunitive Response to Errors* (W = 0.943, *p* = 0.010), and *Patient Safety Grade* (W = 0.856, *p* < 0.001). All other dimensions demonstrated normal distributions according to the Shapiro–Wilk test (*p* > 0.05). The Kolmogorov–Smirnov test was more conservative, suggesting non-normal distributions for ten dimensions, with only *Frequency of Events Reported* (D = 0.101, *p* = 0.200) and *Management Support for Patient Safety* (D = 0.118, *p* = 0.051) showing normal distributions.

For the *control group* (n = 18), the Shapiro–Wilk test revealed non-normal distributions for *Supervisor/Manager Expectations* (W = 0.887, *p* = 0.034), *Organizational Learning–Continuous Improvement* (W = 0.881, *p* = 0.027), *Management Support for Patient Safety* (W = 0.890, *p* = 0.038), *Frequency of Events Reported* (W = 0.881, *p* = 0.027), and *Patient Safety Grade* (W = 0.807, *p* = 0.002). The remaining dimensions followed normal distributions according to the Shapiro–Wilk test. The Kolmogorov–Smirnov results for the *control group* suggested non-normal distributions for *Organizational Learning–Continuous Improvement* (D = 0.229, *p* = 0.014), *Management Support for Patient Safety* (D = 0.209, *p* = 0.037), *Frequency of Events Reported* (D = 0.271, *p* = 0.001), and *Patient Safety Grade* (D = 0.260, *p* = 0.002). The results suggested that several dimensions violated normality assumptions, particularly in the *control group*, which justified the use of non-parametric statistical methods for subsequent analyses in this study.

Th sample characteristics of the responders for both the *intervention group* and the *control group* are reported in detail in Appendix B, Table A3. In Table 1, we report the descriptive statistical measures for each of the 12 composite dimensions. The responses to the two single-item dimensions are reported in Appendix B, Table A4, Table A5, Table A6 and Table A7.

### 3.1. Response Rates and Sample Characteristics

The response rates in the *intervention group* and *control group* in the first measurement were 58% (IG) and 45% (CG), respectively. The post-intervention response rates were 53% (IG) and 48% (CG). In both measurement instances, in both IG and CG, only medical doctors and nurses responded to the survey, while none of the physical therapists participated in the research. Our analyses revealed no significant differences between the *intervention group* and the *control group* in terms of professional roles, years of experience, or work hours. All healthcare providers reported being directly involved in patient care. Most of the healthcare providers in both groups had been working in their respective professions for 6–10 years, both in their department and at this hospital. Furthermore, the vast majority of healthcare providers reported working 60–79 h weekly. Further details are listed in Table A3 in the Appendix B.

### 3.2. Patient Safety Culture Prior to the Intervention

-Primary Outcome Measure-

#### 3.2.1. Patient Safety Culture in the Surgical ICU–Intervention Group Prior to the Intervention

Neither dimension of PSC was assessed as a “strength” in the *intervention group* prior to the intervention and two dimensions were measured as “weaknesses”: *Management Support for Patient Safety* (M 2.7 (min 1.00, max 4.70)) and *Nonpunitive Response to Errors* (M 2.7 (min 1.30, max 5.00)). In response to the single-item question *Patient Safety Grade*, none of the respondents graded it as “failing”, while most of the subjects chose the grade “very good” (44.6%). When responding to the question of how many safety reports they had sent in the past 12 months, the second single-item question in the HOSPSC, most of the responders (72.6%) chose “no reports”.

#### 3.2.2. Patient Safety Culture in the Medical ICU–Control Group Prior to the Intervention

The dimensions *Teamwork Within Units* (M 4.0 (min 2.75, max 5.00)), *Overall Perceptions of Patient Safety* (M 4.15 (min 2.80, max 5.00)), and *Frequency of Events Reported* (M 4.0 (min 1.00, max 5.00)) were valued as “strengths” in the *control group*. The only dimension valued as a “weakness” in the *control group* was *Staffing* (M 2.80 (min 1.30, max 4.00)). In response to the single-item question *Patient Safety Grade*, none of the respondents graded it as “failing,” while most of the subjects chose the grade “very good” (50%). When responding to the single-item question *Number of Events Reported*, the second single-item question in the HOSPSC, most of the responders (57.9%) chose “no reports”.

#### 3.2.3. Differences in Patient Safety Culture Between the Surgical ICU–Intervention Group and the Medical ICU–Control Group in the First Measurement (Inter-Department Differences)

**Figure 2:**
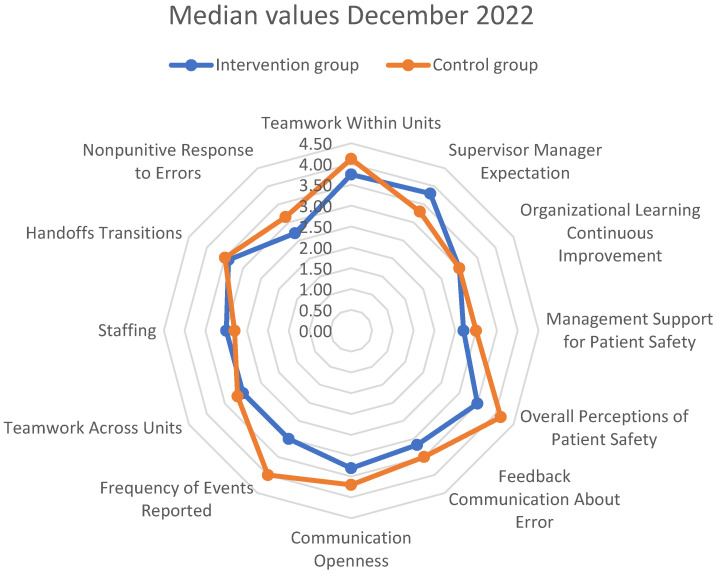
Differences in measured median values of dimensions of *patient safety culture* between the *surgical ICU—intervention group* and the *medical ICU—control Group* in the first measurement (inter-department differences).

As depicted in Figure 2, the first measurements (December 2022) showed better values in most dimensions of *patient safety culture* in the *control group*. Using the conventional significance threshold (*p* < 0.05), the difference was significantly better in the *control group* for three dimensions: *Teamwork Within Units* (Mann–Whitney U test, U = 404.5 *p* = 0.038, r = −0.230 [small to medium effect]), *Overall Perceptions of Patient Safety* (Mann–Whitney U test, U = 393.5 *p* = 0.028, r = −0.244 [small to medium effect]), and *Frequency of Events Reported* (Mann–Whitney U test, U = 2341.5 *p* = 0.024, r = −0.250 [small to medium effect]). After applying the Bonferroni correction to control for multiple comparisons (adjusted α = 0.025), the *Frequency of Events Reported* dimension remained statistically significant, while *Overall Perceptions of Patient Safety* was close to statistical difference. However, the consistent pattern of effects and their practical significance (as indicated by effect sizes ranging from r = −0.230 to r = −0.250) provided additional context beyond statistical significance alone.

When considering the single-item questions, the only significant difference was in response to *Number of Events Reported*, in favor of the answer “no reports” in the *intervention group* (χ^2^(4) = 8.39 *p* = 0.039, Fisher’s exact test).

### 3.3. ICU-DCR Fidelity of Use

-Secondary Outcome Measure-

When analyzing the data from digital hospital records, we found a total of 1663 admissions and discharges to the *surgical ICU* in 2023. As *ICU discharge lists* were supposed to be recorded for each discharge, and we found a total of 1093 discharge lists saved in the digital hospital records, the fidelity in the use of the *ICU discharge readiness checklist* in the *surgical ICU—intervention group* during 2023 was 65.7%.

### 3.4. Patient Safety Culture Following the Intervention

-Tertiary Outcome Measure-

#### 3.4.1. Patient Safety Culture in the Surgical ICU–Intervention Group Following the Intervention

In the follow-up measurement in the surgical ICU, only one dimension of PSC improved enough to be graded as a “strength”: *Teamwork Within Units* (M 4.00 (min 2.50, max 5.00)). Neither dimension was identified as a weakness.

#### 3.4.2. Patient Safety Culture in the Medical ICU–Control Group Following the Intervention

No “strengths” were identified in the follow-up measurements in the *control group*. Three dimensions of PSC were identified as “weaknesses”: *Management Support for Patient Safety* (M 2.7 (min 1.00, max 5.00)), *Staffing* (M 2.8 (min 1.00, max 3.50)), and *Nonpunitive Response to Errors* (M 2.70 (min 1.00, max 3.70)).

#### 3.4.3. Changes in Patient Safety Culture Following the Intervention

##### Changes in Patient Safety Culture in the Surgical ICU–Intervention Group Following the Intervention (Intra-Department Changes)

**Figure 3:**
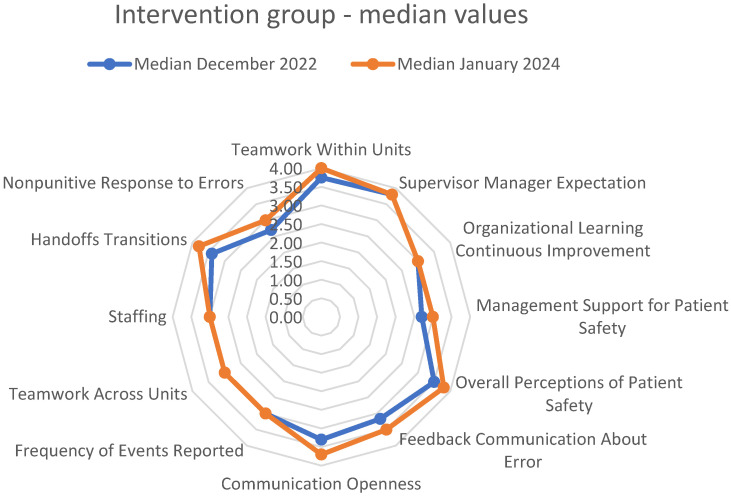
Changes in median values of dimensions of *patient safety culture* following the intervention of implementing ICU-DRC in the surgical ICU (intra-department changes).

The changes measured in the *intervention group* are presented in Table 1 and depicted in Figure 3. Overall, there was a trend toward better results in the follow-up measurements for almost all dimensions measured. However, statistical significance was reached only for the *Teamwork Within Units* dimension, both using the standard significance threshold (*p* < 0.05) and after applying the Bonferroni correction (Mann–Whitney U test, U = 1272.5, *p* = 0.012), r = −0.232 [small to medium effect]).

##### Changes in Patient Safety Culture in the Medical ICU–Control Group Following the Intervention (Intra-Department Changes)

**Figure 4:**
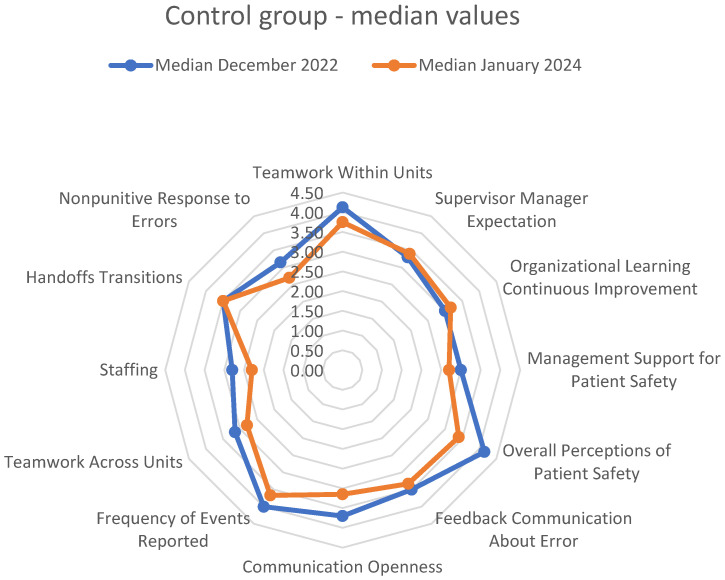
Changes in median values of dimensions of *patient safety culture* in the *medical ICU–control group* (no intervention, intra-department).

PSC measured in the follow-up measurement in the *control group* is presented in Table 1 and depicted in Figure 4. Overall, there was a trend toward worse results in the follow-up measurements for almost all dimensions measured. The negative changes reached statistical significance in three dimensions: *Overall Perceptions of Patient Safety* (Mann–Whitney U test U = 112, *p* = 0.028, r = −0.307 [medium effect]), *Staffing* (Mann–Whitney U test U = 120, *p* = 0.047, r = −0.307 [medium effect]), and *Nonpunitive Response to Errors* (Mann–Whitney U test U = 97.5, *p* = 0.008, r = −0.420 [medium to large effect]). After applying the Bonferroni correction to control for multiple comparisons (adjusted α = 0.025), *Nonpunitive Response to Errors* remained significant, while *Overall Perceptions of Patient Safety* was close to statistical difference. However, the consistent pattern of effects and their practical significance (as indicated by effect sizes ranging from r = −0.307 to r = −0.42) provided additional context for the changes. The following dimensions of *patient safety culture*, which were previously measured as “strengths,” now became “neutral” or “weaknesses”: *Teamwork Within Units*, *Overall Perceptions of Patient Safety*, and *Frequency of Events Reported*. Furthermore, three dimensions that were previously reported as “neutral” became “weaknesses” in the follow-up measurement: *Management Support for Patient Safety*, *Teamwork Across Units*, and *Nonpunitive Response to Errors*.

##### Differences in Patient Safety Culture Between the Surgical ICU–Intervention Group and Medical ICU–Control Group in the Follow-Up Measurement (Inter-Department Differences)

**Figure 5:**
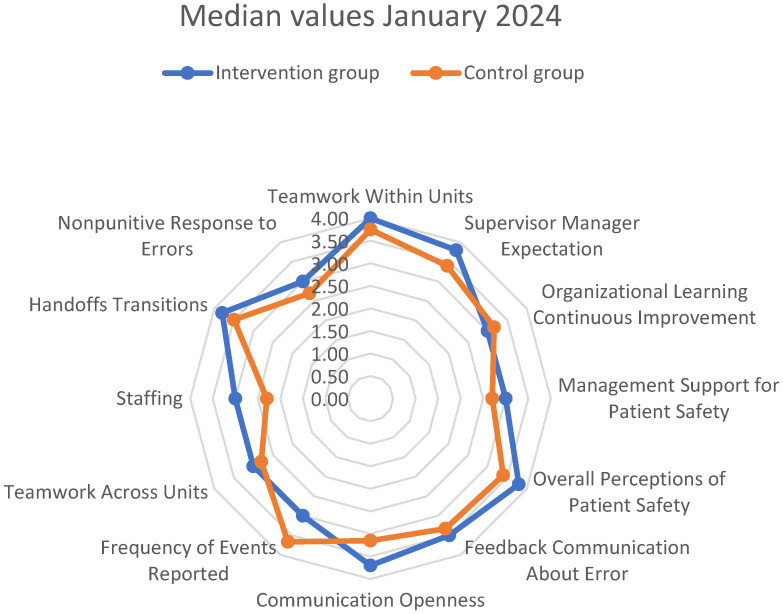
Differences in measured median values of dimensions of *patient safety culture* between the *surgical ICU–intervention group* and *medical ICU–control group* in the second measurement (inter-department changes).

An observable pattern of improvement was measured in the follow-up measurement in the *intervention group*, and lower scores were measured in the *control group*. The cumulative effect of these contrasting trends resulted in the *intervention group* outperforming the *control group* in almost all dimensions at follow-up, as depicted in Figure 5. Hence, in the follow up measurement, four dimensions scored significantly higher in the *intervention group* than in the *control group*:

1. *Supervisor/Manager Expectations* (Mann–Whitney U test U = 357.5 *p* = 0.016, r = −0.276 [small to medium effect]).
2. *Management Support for Patient Safety* (Mann–Whitney U test, U = 371.5 *p* = 0.025, r = −0.257 [small to medium effect].
3. *Staffing* (Mann–Whitney U test, U = 257.5 *p* < 0.001, r = −0.412 [medium to large effect]).
4. *Nonpunitive Response to Errors* (Mann–Whitney U test, U = 530, *p* < 0.001, r = −0.328 [medium effect]).

## 4. Discussion

When analyzing data from two Croatian hospitals, the authors of previous studies found that 9 dimensions had values ≥3, which was deemed a satisfactory level of PSC [55,67]; the dimensions of *Staffing*, *Nonpunitive Response to Errors*, and *Hospital Management Support for Patient Safety* [55] scored lower. Our results in the *surgical ICU*, prior to the intervention, also showed that the same three dimensions scored ≤ 3. The first measurements in the *medical ICU* showed even better PSC, with only the dimension of *Staffing* being graded ≤ 3.

The observable patterns of improvement in PSC in the *intervention group* resulted in all dimensions of PSC being graded ≥3. Moreover, the *Teamwork within Units* dimension had statistically significantly higher results after the intervention, with small to medium effect. Surprisingly, the results in the *medical ICU–control group* did not stagnate, as one might expect, but deteriorated in the follow-up measurement. Thus, the following dimensions of PSC became weaknesses: *Teamwork Across Units*, *Staffing*, and *Nonpunitive Response to Errors*. The *Nonpunitive Response to Errors* dimension demonstrated statistically significantly lower results with a medium to large effect in the follow-up measurement.

It is also interesting to note a discrepancy in the results obtained for the composite dimension *Overall Perceptions of Safety* and the single-item measure *Patient Safety Grade*, which address a similar concept in different ways. In both measurements in both the *surgical ICU* and *medical ICU*, most respondents gave a “very good” response when asked for a *Patient Safety Grade*. On the other hand, the dimension *Overall Perceptions of Safety* was consistently graded below 4, except for the first measurement in the *medical ICU*. While a previous study found these two dimensions to be congruent, they did postulate that such discrepancies may stem from a dominant *culture of blame* preventing participants from expressing themselves completely honestly [60].

Comparing the results obtained in this study with previous findings regarding PSC in ICUs is challenging for two main reasons: there is limited previous research focused solely on *safety culture* in adult ICUs [68,69,70], and there are a multitude of instruments used to assess PSC and even more ways to present or interpret the data obtained [36]. However, our results showed that the baseline measurements in both ICUs tested were better than previous research suggested, as Tlili et al. found in a multicenter ICU study that neither dimension scored above 50%, i.e., all dimensions were deemed “in need of improvement” [69]. Huang et al. [70] used a different PSC assessment tool, the Safety Attitudes Questionnaire (SAQ) [71], making direct comparisons impossible, yet they reached a similar conclusion that *safety culture* “scores were mostly low to moderate, and varied widely across ICUs” [70]. On the other hand, the PSC measurements in Neonatal Intensive Care Units (NICUs) were mostly positive, even though the results varied significantly between individual NICUs [72].

It is also impossible to directly compare the change in PSC following the introduction of the ICU-DRC to previous studies as none have been published to date, to the best of our knowledge. However, the World Health Organization Surgical Safety Checklist [51,73], a widely adopted similar instrument applied in perioperative medicine, is believed to have yielded a positive impact on patient outcomes [51,74,75,76] primarily through enhancing *safety* culture [77]. Several studies have demonstrated this positive effect [78,79,80], yet there is some contradicting evidence available as well [33,81]. In one recent study testing the longitudinal effects of WHO SSC implementation, the authors found significant improvements in a majority of *safety culture* dimensions from 2009 to 2017 after the WHO SSC was imbedded into a broader *National Patient Safety Program* in Norway [52]. Haugen et al. suggested that improved PSC over time following the implementation of the WHO SSC might partly be explained by “boards explicitly prioritizing quality improvement, balancing short and long-term investment in quality improvement, using data for quality improvement, engaging staff and patients in quality improvement and encouraging a culture of continuous improvement” and the WHO SSC being a part of a comprehensive safety program [52,53]. Our intervention was not a part of a broader strategic safety campaign but rather an isolated early clinical adaptation of a focused, scientifically sound list of criteria [28] envisioned to aid in the discharge process in the ICU. Furthermore, in their 2020 study, Haugen et al. found a significant correlation between an increase in fidelity of application of the WHO SSC and increased scores for dimensions of *safety culture*, as compliance with the application of the WHO SSC in practice rose over time [52]. During the one-year period of our intervention, we achieved 65.7% compliance with the use of the ICU-DCR, substantially less than the 75% and 88% in 2009 and 2017, respectively, reported by the Norwegian group of authors with the implementation of the WHO SSC [52]. Hopefully, in time, focused ICU team education on the subject matter of both patient safety and proper discharge processes and managerial insistence on proper procedure will increase compliance with the ICU-DRC, and this, in turn, could be associated with further positive effects on *patient safety culture* [82,83].

Even though only one dimension in the *intervention group* improved enough for the change to be statistically significant with a small to medium effect (intra-department), a total of four different dimensions (*Supervisor Manager Expectations*, *Management Support for Patient Safety*, *Staffing*, and *Nonpunitive Response to Errors*) tested significantly higher in the *intervention group* than in the *control group* in the follow-up measurement (inter-department) with a small to medium effect. These results suggested that implementing a structured discharge process not only addresses the specific transition point in care but may also be correlated with protective mechanisms against broader deterioration in *patient safety culture* during periods of organizational change. The consistent pattern of improvement across dimensions highlights the association of targeted safety with an enhancement in the overall *safety culture* within intensive care environments. Vikan et al. found that increased PSC scores were related to reduced AE rates in 76% of studies included in their scoping review from 2023 [40]. In particular, increased scores in dimensions pertaining to the managerial role in shaping the *safety culture*—which happen to be three of the four dimensions that were significantly better in the *intervention group* than in the *control group* in the follow-up measurement—were positively associated with fewer AEs [40,69,84,85,86,87,88], which is in line with the previously described key role of senior leadership [89]. On the other hand, when analyzing the responses to dimensions pertaining to teamwork, we surprisingly did not demonstrate a significant improvement in either *Handoffs and Transitions* or in the dimension of *Teamwork Across Units*, the areas of *safety culture* toward which the intervention was specifically directed and which have also been shown to substantially positively affect patient outcomes [85]. While the intervention was positively associated with improvements in *Teamwork Within Units*, a dimension of PSC clearly linked with decreased AE rates [85,86,87,88,90], it is unclear whether concurrent initiatives or contextual factors contributed to this improvement.

### Limitations and Strengths

Our study has several key limitations. While the study was not randomized, our quasi-experimental design represents the most rigorous approach possible given the nature of the intervention. The complexity of implementing the ICU-DRC across an entire operational unit precluded the randomization of individual clinicians or patients, as the intervention inherently required unit-wide workflow modifications and team-based implementation. However, we acknowledge the baseline differences between the surgical and medical ICUs in terms of staffing ratios (106 vs. 42 providers), patient throughput (1663 vs. 867 annual admissions), case mix complexity, and subspecialty focus. These structural and operational differences likely contributed to the baseline variation in PSC measurements, as evidenced by the higher initial scores in the control group for the dimensions Teamwork Within Units, Overall Perceptions of Patient Safety, and Frequency of Events Reported. These pre-existing differences represent an inherent limitation of our comparative analysis, although the longitudinal within-group changes remained methodologically robust.

Furthermore, we acknowledge several limitations related to sample size and potential non-response bias. Our response rates (53–58% in the intervention group and 45–48% in the control group) reflect typical participation in healthcare safety surveys [52,53,55,56,91,92] but introduce the possibility of non-response bias. While the demographic analysis of respondents, presented in Table A3, revealed no significant differences between the intervention group and control group in terms of professional roles, years of experience, or work hours, we cannot rule out that respondents and non-respondents may have differed in either their demographic and professional characteristics or their perceptions of patient safety culture. As the non-respondents did not answer any part of the survey, data regarding their characteristics are unavailable for comparison. This limitation is partially mitigated by our longitudinal approach, as we detected no significant differences in any of the professional or demographic data across measurement points, which might suggest consistent representation. However, this does potentially limit the generalizability of our findings, as regards both internal and external validity.

Furthermore, regarding external validity, our focus on two specialized ICUs within a single tertiary teaching hospital limits generalizability to other settings, making our findings most applicable to similar medical centers with comparable organizational structures.

We achieved a checklist fidelity of 65.7%, which aligns with the lower end of reported adherence rates to similar interventions [52]. Notably, this outcome was achieved despite the checklist being mandatory for use. Although a recent study showed a linear relationship between fidelity and treatment effect size, there was no specific cut-off fidelity rate below which an intervention would definitely be ineffective [93]. A further contextual factor to be considered is the implementaion of the ICU-DRC being an isolated safety intervention rather than part of a broader organizational safety initiative. This contrasts with studies where checklists like the WHO Surgical Safety Checklist were embedded within comprehensive quality improvement programs, which often yielded stronger cultural shifts [52]. The absence of complementary strategies (e.g., leadership engagement, staff training) may have diluted the intervention’s effect, particularly in dimensions linked to managerial support and nonpunitive error response. Potential additional confounding variables could stem from the study period coinciding with hospital renovations, introducing uncertainty about ICU occupancy and workload distribution. While unmeasured, these external factors could have influenced both fidelity to intervention in the intervention group and staff perceptions of patient safety culture in both groups. Additionally, the lack of paired respondent tracking precluded individual-level longitudinal analysis, relying instead on anonymized aggregate data.

This study possesses several strengths that enhance the validity and impact of its findings. First, it addresses a critical issue in healthcare by focusing on handoffs and transitions in care, recognized as high-risk periods, thus targeting a key area for improvement. The proactive implementation and evaluation of the ICU-DRC as a tool to improve patient safety by ensuring discharge readiness further strengthens the study’s practical relevance. The selection of two specialized ICUs within the same hospital offered several methodological advantages, including exposure to identical institutional leadership, hospital-wide policies, and accreditation standards. The study’s methodology was robust, as the controlled intervention design allowed for a comparison between an intervention group and a control group, providing a basis for assessing the impact of the ICU-DRC. The use of the validated instrument, the HSOPSC, ensured the reliable and quantitative measurement of *patient safety culture*. The study defined clear and measurable outcomes to assess the impact of the ICU-DRC on PSC. The fact that the Croatian version of the HOSPSC has been used for over 10 years and is deemed adequate for secondary and tertiary levels of care bolsters the validity of the PSC measurements. Furthermore, the study’s execution in a real-world setting within a tertiary teaching hospital enhances its ecological validity and the generalizability of the results [94]. Finally, the acceptable response rates achieved in both the *intervention* and *control groups* contribute to the reliability and representativeness of the collected data.

## 5. Conclusions

Implementing the ICU-DRC as an isolated safety intervention aimed at optimizing ICU patient throughput was associated with an observable pattern toward better PSC outcomes in the *intervention group*, resulting from divergent trajectories between the *intervention* and *control groups*. While the *intervention group* exhibited statistical improvement in one dimension (*Teamwork Within Units*) and consistent improvements across multiple PSC dimensions after the application of the ICU-DRC, the *control group*, by comparison, experienced deterioration in several areas (intra-department changes). In the follow-up measurement, the *intervention group* demonstrated significantly better outcomes than the *control group* in organizational and leadership-focused dimensions, with *Staffing* showing the largest effect, followed by *Nonpunitive Response to Errors* (inter-department changes). The significant enhancements in *Supervisor/Manager Expectations* and *Management Support for Patient Safety* further confirmed the positive association of implementing the ICU-DRC with leadership engagement with safety practices. Even though the intervention was not part of a broader comprehensive safety initiative, the associated positive effects across a relatively short time period imply its long-term potential in changing the *safety climate*, thus helping to reduce the risk of *adverse events* for patients treated in the ICU. Importantly, our results indicate that such interventions are not merely administrative burdens but can be linked with tangible benefits.

Future studies should consider larger multi-center studies with extended follow-up periods to evaluate sustainability. Additionally, investigating the relationship between implementation fidelity and outcomes, examining the integration of discharge checklists into comprehensive safety programs, and evaluating differential effects across varying ICU contexts would provide valuable insights. However, randomization may not always be feasible within healthcare improvement contexts due to ethical considerations, logistical constraints, and the need for the timely implementation of safety interventions. By continuing to explore and refine these interventions, we can better understand how they contribute to a robust *patient safety culture* and ultimately improve patient outcomes.

## Figures and Tables

**Table 1:** Intra-department values and statistically significant differences across 12 PSC dimensions in the *intervention (surgical ICU)* and *control (medical ICU) groups* before (December 2022) and after (January 2024) the intervention.

		Dimensions of *Patient Safety Culture*							
		Median Values							
		Surgical ICU				Medical ICU			
		December 2022	January 2024	Δ *	Mann–Whitney U test	December 2022	January 2024	Δ *	Mann–Whitney U test
**D1**	**Teamwork Within Units**	3.75 (min 2.00, max 5.00)	4.00 (min 2.50, max 5.00)	**0.25**	U = 1272.5, *p* = 0.012, r = −0.232 (small to medium effect)	4.13 (min 2.75, max 5.00)	3.75 (min 2.50, max 5.00)	*−0.38*	
**D2**	**Supervisor/Manager Expectations**	3.80 (min 1.80, max 5.00)	3.80 (min 1.80, max 5.00)	0.00		3.30 (min 2.50, max 4.80)	3.40 (min 1.50, max 5.00)	**0.10**	
**D3**	**Organizational Learning–Continuous Improvement**	3.00 (min 1.33, max 4.33)	3.00 (min 1.67, max 4.33)	0.00		3.00 (min 2.00, max 3.67)	3.17 (min 2.33, max 4.33)	**0.17**	
**D4**	**Management Support for Patient Safety**	2.70 (min 1.00, max 4.70)	3.00 (min 1.00, max 4.70)	**0.30**		3.00 (min 2.00, max 4.30)	2.70 (min 1.00, max 5.00)	*−0.30*	**U = 122.5, *p* = 0.056, (Bonferroni-corrected α = 0.025)** **r = −0.307 (medium effect)**
**D5**	**Overall Perceptions of Patient Safety**	3.50 (min 1.50, max 5.00)	3.80 (min 1.50, max 4.80)	**0.30**		4.15 (min 2.80, max 5.00)	3.40 (min 1.80, max 5.00)	*−0.75*	U = 112, *p* = 0.028, (Bonferroni-corrected α = 0.025) r = −0.353 (medium effect)
**D6**	**Feedback Communication About Errors**	3.17 (min 1.00, max 5.00)	3.50 (min 1.00, max 5.00)	**0.33**		3.50 (min 1.67, max 4.67)	3.33 (min 1.67, max 5.00)	*−0.17*	
**D7**	**Communication Openness**	3.30 (min 1.70, max 4.70)	3.70 (min 1.30, max 5.00)	**0.40**		3.70 (min 1.30, max 4.70)	3.15 (min 2.00, max 5.00)	*−0.55*	
**D8**	**Frequency of Events Reported**	3.00 (min 1.00, max 5.00)	3.00 (min 1.00, max 5.00)	0.00		4.00 (min 1.00, max 5.00)	3.67 (min 1.33, max 5.00)	*−0.33*	
**D9**	**Teamwork Across Units**	3.00 (min 1.80, max 4.80)	3.00 (min 2.00, max 4.50)	0.00		3.15 (min 1.80, max 4.50)	2.80 (min 1.30, max 5.00)	*−0.35*	
**D10**	**Staffing**	3.00 (min 1.30, max 5.00)	3.00 (min 1.30, max 4.80)	0.00		2.80 (min 1.30, max 4.00)	2.30 (min 1.00, max 3.50)	*−0.50*	U = 120, *p* = 0.047, (Bonferroni-corrected α = 0.025), r = −0.318 (medium effect)
**D11**	**Handoffs and Transitions**	3.40 (min 2.00, max 5.00)	3.80 (min 2.00, max 5.00)	**0.40**		3.50 (min 2.00, max 5.00)	3.50 (min 2.30, max 5.00)	0.00	
**D12**	**Nonpunitive Response to Errors**	2.70 (min 1.30, max 5.00)	3.00 (min 1.30, max 5.00)	**0.30**		3.15 (min 1.70, max 5.00)	2.70 (min 1.00, max 3.70)	*−0.45*	U = 97.5, *p* = 0.008, r = −0.420 (medium to large effect)

* Values in bold represent a positive change in values, while values in italics represent a negative change in values.

## Data Availability

Data are available upon reasonable request from the corresponding author.

## Abbreviations

The following abbreviations are used in this manuscript

AEs	Adverse events
ICU	Intensive care unit
ICU-DRC	Intensive care unit discharge readiness checklist
IG	Intervention group
CG	Control group
HSOPSC	Hospital Survey on Patient Safety Culture
M	Median value
IQR	Interquartile range
PSC	Patient safety culture
SSC	Surgical safety checklist
WHO	World Health Organization

## Appendix A. Materials and Methods

**Table A1:** Dimensions of safety culture are formed from the 42 questions of the HOSPSC [37,54].

The 12 Safety Culture Dimensions of the US Hospital Survey on *Patient Safety Culture*	
Safety Culture Dimensions	Items
**D1** **Teamwork Within Units**	A1 People support one another in this unit
	A3 When a lot of work needs to be done quickly, we work together as a team to get the work done
	A4 In this unit, people treat each other with respect
	A11 When one area in this unit gets really busy, others help out
**D2** **Supervisor/Manager Expectations**	B1 My supervisor/manager says a good word when he/she sees a job done according to established patient safety procedures
	B2 My supervisor/manager seriously considers staff suggestions for improving patient safety
	B3r Whenever pressure builds up, my supervisor/manager wants us to work faster, even if it means taking shortcuts
	B4r My supervisor/manager overlooks patient safety problems that happen over and over (reverse worded)
**D3** **Organizational Learning–Continuous Improvement**	A6 We are actively doing things to improve patient safety
	A9 Mistakes have led to positive changes here
	A13 After we make changes to improve patient safety, we evaluate their effectiveness
**D4** **Management Support for Patient Safety**	F1 Hospital management provides a work climate that promotes patient safety
	F8 The actions of hospital management show that patient safety is a top priority
	F9r Hospital management seems interested in patient safety only after an adverse event happens (reverse worded)
**D5** **Overall Perceptions of Patient Safety**	A15 Patient safety is never sacrificed to get more work done
	A18 Our procedures and systems are good at preventing errors from happening
	A10r It is just by chance that more serious mistakes don’t happen around here (reverse worded)
	A17r We have patient safety problems in this unit (reverse worded)
**D6** **Feedback Communication About Errors**	C1 We are given feedback about changes put into place based on event reports
	C3 We are informed about errors that happen in this unit
	C5 In this unit, we discuss ways to prevent errors from happening again
**D7** **Communication Openness**	C2 Staff will freely speak up if they see something that may negatively affect patient care
	C4 Staff feel free to question the decisions or actions of those with more authority
	C6r Staff are afraid to ask questions when something does not seem right (reverse worded)
**D8** **Frequency of Event Reporting**	D1 When a mistake is made, but is caught and corrected before affecting the patient, how often is this reported?
	D2 When a mistake is made, but has no potential to harm the patient, how often is this reported?
	D3 When a mistake is made that could harm the patient, but does not, how often is this reported?
**D9** **Teamwork Across Units**	F4 There is good cooperation among hospital units that need to work together
	F10 Hospital units work well together to provide the best care for patients
	F2r Hospital units do not coordinate well with each other (reverse worded)
	F6r It is often unpleasant to work with staff from other hospital units (reverse worded)
**D10** **Staffing**	A2 We have enough staff to handle the workload
	A5r Staff in this unit work longer hours than is best for patient care (reverse worded)
	A7r We use more agency/temporary staff than is best for patient care (reverse worded)
	A14r We work in ‘crisis mode’, trying to do too much, too quickly (reverse worded)
**D11** **Handoffs and Transitions**	F3r Things “fall between the cracks” when transferring patients from one unit to another (reverse worded)
	F5r Important patient care information is often lost during shift changes (reverse worded)
	F7r Problems often occur in the exchange of information across hospital units (reverse worded)
	F11r Shift changes are problematic for patients in this hospital (reverse worded)
**D12** **Nonpunitive Response to Errors**	A8r Staff feel like their mistakes are held against them (reverse worded)
	A12r When an event is reported, it feels like the person is being written up, not the problem (reverse worded)
	A16r Staff worry that mistakes they make are kept in their personnel file (reverse worded)
**Patient Safety Grade ***	E1 Please give your work area/unit in this hospital an overall grade on patient safety
**Number of Events Reported †**	G1 In the past 12 months, how many event reports have you filled out and submitted?

* Single-item measure—grades A through E as response category. † Single-item measure—numeric response categories.

**Table A2:** The criteria used to assess ICU discharge readiness, as described by Hiller et al. [28].

	Final List of ICU Discharge Criteria	Fit for ICU Discharge	Needs Further Intensive Care Therapy/Monitoring	Value Calculation Method to Evaluate Discharge Readiness	Criterion Importance Rank	Exceptions When “Mandatory to Be Met”
Respiratory system						
**1**	Is the patient’s (own/artificial) airway patent?	yes	no	n.a	mandatory to be met	EOL-care, agreed treatment limits
**2**	Is the cough effective in a way that the patient can be handled at the receiving unit?	yes	no	n.a	Mandatory to be met	EOL-care, agreed treatment limits, p. with low level of consciousness and requiring long-term artificial airway aspiration
**3**	Blood oxygenation: Stable SpO2 (≥ 92% OR stable around lower patient individual baseline value) AND patient is breathing room air?	yes	no	Worst value must be above threshold value AND trend must be stable over defined time frame	Mandatory to be met	EOL-care, agreed treatment limits, p. with chronic lung disease
**4**	Respiratory rate: Stable RR trend with 10 ≤ resp ≤ 30 (pm) OR patient’s individual baseline value is met?	yes	no	Worst value must be within acceptable range AND trend must be stable over defined time frame	Mandatory to be met	EOL-care
**5**	If further respiratory support is needed, feasible at the receiving unit?	yes	no	n.a	Mandatory to be met	
**6**	If the patient is in need of tracheal suctioning, feasible at the receiving unit?	yes	no	n.a	Mandatory to be met	
**7**	If the patient is in need of long-term tracheostomy, could adequate care be provided at the receiving unit?	yes	no	n.a	Mandatory to be met	If patient is sufficiently trained and capable to take care of the tracheostomy
**Cardiovascular system**						
**8**	Heart rate: Stable HR trend with 50 ≤ hr ≤ 110 (bpm) OR patient’s individual baseline value is met?	yes	no	Worst value must be within acceptable range AND trend must be stable over defined time frame	Mandatory to be met	EOL-care, agreed treatment limits, pre-existing bradycardia
**9**	Cardiac rhythm: Stable cardiac rhythm OR tolerable intermittent arrhythmia over defined time frame?	yes	no	Trend must be stable over defined time frame	Mandatory to be met	EOL-care, agreed treatment limits, p. has intermittent AF but is otherwise hemodynamically stable with controlled ventricular response
**10**	Mean arterial pressure: Stable MAP trend with 60 < map ≤ 110 (mmHg) OR patient’s individual baseline value is met?	yes	no	Worst value must be within acceptable range AND trend must be stable over defined time frame	Mandatory to be met	EOL-care, agreed treatment limits
**11**	Hypervolemia/hypovolemia: Does the current volemia status require ICU monitoring?	no	yes	n.a	Mandatory to be met	
**12**	Hemoglobin value stable over defined time frame?	yes	no	Trend must be stable over defined time frame	Mandatory to be met	EOL-care, agreed treatment limits
**13**	Significant active bleeding or high risk of significant bleeding?	no	yes	n.a	Mandatory to be met	EOL-care, agreed treatment limits
**14**	If the patient needs continued monitoring at the receiving unit, are required technology/staff capabilities in place?	yes	no	n.a	Mandatory to be met	
**15**	If the patient carries a percutaneous transient pacemaker, could that be handled at the receiving unit?	yes	no	n.a	Mandatory to be met	
**16**	If low-dose vasoactives are in use, is the patient manageable at the receiving unit?	yes	no	n.a	Mandatory to be met	
**Central nervous system**						
**17**	Can the neurological status of the patient be adequately handled and monitored at the receiving unit?	yes	no	n.a	Mandatory to be met	
**Pain**						
**18**	Pain therapy sufficient and feasible at the receiving unit?	yes	no	n.a	Mandatory to be met	
**Urogenital system**						
**19**	Do urine output, electrolyte level, and renal function allow patient discharge?	yes	no	n.a	Mandatory to be met	EOL-care, agreed treatment limits
**20**	If required, is renal replacement therapy possible outside the ICU?	yes	no	n.a	Mandatory to be met	EOL-care, agreed treatment limits
**Fluid loss and drainage**						
**21**	Could fluid loss or drainage(s) be monitored and handled adequately at the receiving unit?	yes	no	n.a	Mandatory to be met	
Medication and nutrition						
**22**	If the patient needs continuous IV application (e.g., insulin, glucose, antibiotics, vasopressors, nutrition), allowed and feasible at the receiving unit?	yes	no	n.a	mandatory to be met	
Patient diagnosis, prognosis, and preferences						
**23**	Patient’s preference is to stop intensive care therapy and to leave the ICU	yes	no	n.a	Not mandatory to be met	
**24**	Therapeutic susceptibility: Patient does not benefit from ICU care anymore and negative effects may outweigh	yes	no	n.a	mandatory to be met	
Institution-specific criteria						
**25**	Patient no longer meets ICU admission criteria and meets admission criteria for a lower level of care	yes	no	n.a	mandatory to be met	
**26**	Do current acuity and dependency levels and current workload at the receiving unit allow to admit and take care of this patient?	yes	no	n.a	mandatory to be met	
**27**	If the patient is immune compromised or infectious, could the patient be handled and adequately cared for at the receiving unit?	yes	no	n.a	mandatory to be met	
**28**	If discharge at night or weekend cannot be avoided, are measures in place to protect patient safety?	yes	no	n.a	mandatory to be met	EOL-care, agreed treatment limits, prioritarisation against patients with greater need for intensive care

## Appendix B. Results

**Table A3:** Respondents’ characteristics.

	Intervention Group		Control Group	
**Survey time**	December 2022	January 2024	December 2022	January 2024
	**n = 62**	**n = 56**	**n = 19**	**n = 20**
**Characteristics**				
**Profession**	**n (%)**	**n (%)**	**n (%)**	**n (%)**
Doctor	32 (51.6)	23 (41.1)	3 (15.8)	5 (25)
Nurse	30 (48.4)	33 (58.9)	16 (84.2)	15 (75)
**Years working in the profession**				
<1	2 (3.2)	2 (3.6)	1 (5.2)	2 (10)
1–5	13 (21)	14 (25)	12 (63.1)	9 (45)
6–10	17(27.4)	17 (30.4)	5 (26.3)	6 (30)
11–15	8 (12.9)	6 (10.7)	0	2 (10)
16–20	11 (17.7)	4 (7.1)	0	1 (5)
>21	11 (17.7)	13 (23.2)	1 (5.2)	0
**Years working in this department**				
<1	4 (6.5)	2 (3.6)	3 (15.8)	3 (15)
1–5	12 (19.4)	13 (23.2)	9 (47.4)	7 (35)
6–10	16 (25.8)	18 (32.1)	5 (26.3)	7 (35)
11–15	8 (12.9)	7 (12.5)	1 (5.2)	3 (15)
16–20	12 (19.4)	9 (16.1)	0	0
>21	10 (16.1)	7 (12.5)	1 (5.2)	0
**Years working at this hospital**				
<1	4 (6.5)	2 (3.6)	2 (10.5)	3 (15)
1–5	10 (16.1)	12 (21.4)	10 (52.6)	7 (35)
6–10	18 (29)	17 (30.4)	4 (21.1)	6 (30)
11–15	8 (12.9)	7 (12.5)	2 (10.5)	3 (15)
16–20	9 (14.5)	9 (16.1)	0	1 (5)
>21	13 (21)	9 (16.1)	1 (5.2)	0
**Weekly working hours**				
<20	0	0	0	0
20–39 h a week	1 (1.6)	0	0	0
40–59 h a week	4 (6.5)	8 (14.3)	0	2 (10)
60–79 h a week	52 (83.9)	43 (76.8)	19 (100)	16 (80)
80–99 h a week	4 (6.5)	3 (5.4)	0	2 (10)
>100 h a week	1 (1.6)	2 (3.6)	0	0
**Direct contact with patient**				
Yes	62 (100)	56 (100)	19 (100)	20 (100)
No	0	0	0	0

**Table A4:** Responses to single-item measure—“Patient Safety Grade”—in the first measurement, prior to the intervention.

December 2022				
Patient Safety Grade		Intervention Group	Control Group	Total
**Please give your work area/unit in this hospital an overall grade on patient safety**	Failing	0	0	0
	Poor	2	0	2
	Acceptable	17	3	20
	Very good	25	9	34
	Excellent	12	6	18
**Total**		56	18	74

**Table A5:** Responses to single-item measure—“Patient Safety Grade”—in the second measurement, after the intervention.

January 2024				
Patient Safety Grade		Intervention Group	Control Group	Total
**Please give your work area/unit in this hospital an overall grade on patient safety**	Failing	0	0	0
	Poor	1	2	3
	Acceptable	16	7	23
	Very good	23	8	31
	Excellent	16	3	19
**Total**		56	20	76

**Table A6:** Responses to single-item measure—“*Number of Events Reported*”—in the first measurement, prior to the intervention.

December 2022				
*Number of Events Reported*		Intervention Group	Control Group	Total
**In the past 12 months, how many event reports have you filled out and submitted?**	*No reports*	*45 **	*11*	*56*
	1–2 reports	14	4	18
	3–5 reports	1	4	5
	6–10 reports	1	0	1
	11–20 reports	1	0	1
**Total**		62	19	81

* χ^2^(4) = 8.39 *p* = 0.039, Fisher’s exact test.

**Table A7:** Responses to single-item measure—“*Number of Events Reported*”—in the second measurement, after the intervention.

January 2024				
		Intervention Group	Control Group	Total
**In the past 12 months, how many event reports have you filled out and submitted?**	*None*	44	12	56
	1–2 reports	10	7	17
	3–5 reports	2	1	3
	6–10 reports			
	11–20 reports			
**Total**		56	20	76
